# Porous 3D Scaffolds Enhance MSC Vitality and Reduce Osteoclast Activity

**DOI:** 10.3390/molecules26206258

**Published:** 2021-10-16

**Authors:** Miriam Spreda, Nicole Hauptmann, Veronika Lehner, Christoph Biehl, Klaus Liefeith, Katrin Susanne Lips

**Affiliations:** 1 Experimental Trauma Surgery, Justus-Liebig-University Giessen, Aulweg 128, 35392 Giessen, Germany; Miriam.Spreda@med.uni-giessen.de (M.S.); Veronika.Lehner@chiru.med.uni-giessen.de (V.L.); 2 Department of Biomaterials, Institute for Bioprocessing and Analytical Measurement Techniques e.V. (iba), Rosenhof, 37308 Heilbad Heiligenstadt, Germany; Nicole.Hauptmann@iba-heiligenstadt.de (N.H.); Klaus.Liefeith@iba-heiligenstadt.de (K.L.); 3 Department of Trauma, Hand and Reconstructive Surgery, University Hospital of Giessen-Marburg GmbH, Campus Giessen, Rudolf-Buchheim-Strasse 7, 35392 Giessen, Germany; Christoph.Biehl@chiru.med.uni-giessen.de

**Keywords:** 3D-printing, osteoclasts, mesenchymal stromal cells, tartrate-resistant acid phosphatase, mRNA expression, implant, bone, cartilage, polymer, poly-lactide-caprolactone

## Abstract

In the context of an aging population, unhealthy Western lifestyle, and the lack of an optimal surgical treatment, deep osteochondral defects pose a great challenge for the public health system. Biodegradable, biomimetic scaffolds seem to be a promising solution. In this study we investigated the biocompatibility of porous poly-((D,L)-lactide-ε-caprolactone)dimethacrylate (LCM) scaffolds in contrast to compact LCM scaffolds and blank cell culture plastic. Thus, morphology, cytotoxicity and metabolic activity of human mesenchymal stromal cells (MSC) seeded directly on the materials were analyzed after three and six days of culturing. Further, osteoclastogenesis and osteoclastic activity were assessed using reverse-transcriptase real-time PCR of osteoclast-specific genes, EIA and morphologic aspects after four, eight, and twelve days. LCM scaffolds did not display cytotoxic effects on MSC. After three days, metabolic activity of MSC was enhanced on 3D porous scaffolds (PS) compared to 2D compact scaffolds (CS). Osteoclast activity seemed to be reduced at PS compared to cell culture plastic at all time points, while no differences in osteoclastogenesis were detectable between the materials. These results indicate a good cytocompatibility of LCM scaffolds. Interestingly, porous 3D structure induced higher metabolic activity of MSC as well as reduced osteoclast activity.

## 1. Introduction

Deep osteochondral defects represent an increasing worldwide public health problem caused by the aging population, unhealthy lifestyles, and sport injuries. They impair the public health system as well as the life quality of patients due to deteriorated joint function and pain. Several surgical approaches are available, e.g., autologous or allogenic tissue transplantation [1]. However, the treatment of deep osteochondral defects is still challenging because of the limited self-healing competence of cartilage, the complex cartilage–bone interface, and the requirement of load-bearing devices [2]. Progress in the field of 3D biomaterial generation allows the production of biphasic porous scaffolds that mimics the cartilage, tide zone, and subchondral bone. These biomimetic biphasic porous scaffolds should hold unlimited availability and personalized size adaption [3] capability [4]. Good biocompatibility would let them be the desired surgical option for the treatment of deep osteochondral defects. Thus, one aim of present study was to analyze the cellular compatibility of new designed 3D implants.

Biphasic scaffolds obtained promising results in in vitro studies [2,5,6]. Still, an ideal implant is yet to be developed due to the challenges of mimicking the complexity of osteochondral interface [7].

Despite profound differences in histological appearance and mechanical properties, subchondral bone and articular cartilage form a functional union [8]. Even if it is not always damaged itself, subchondral bone plays an important role in the genesis and outcome of osteochondral defects [8,9,10,11,12]. Therefore, we aimed here to verify the suitability of the osteophase of the new biphasic implant.

Porous scaffolds support tissue ingrowth and increase the stability of implants by biological fixation. The scaffolds should preserve the viability of ingrowing host cells. To anchor the scaffold into the host bone, mesenchymal stromal cells (MSC) as progenitors of the bone forming osteoblasts need first to colonize the scaffold and then to differentiate into active osteoblasts. This requires a good viability of the MSC that we aimed to prove in the present study. The bioactivity of scaffolds can be increased by the used material as well as by the geometric 3D structure [13].

Porous 3D scaffolds are fabricated through a variety of methods, e.g., electrospinning, freeze drying, stereolithography, or melt electrowriting [14,15,16]. In this study, scaffolds were fabricated via 2-photon-polymerization (2-PP) and consisted of poly-((D,L)-lactide-ε-caprolactone)dimethacrylate (LCM). 2-PP allows precise manufacturing of defined geometric structures with tailor-made porosity, pore interconnectivity, and mechanical properties in the nanometer range [14,17,18]. The used materials have to be infrared transparent and highly viscous. Several methacrylated or acrylated synthetic or biological polymers or hybrid materials are qualified, for instance hyaluronic acid, gelatin, polycaprolactone, or poly-lactide [14,18,19,20,21]. Hybrid materials made of poly-lactide (PLA) and poly-caprolactone (PCL) performed well in previous studies [3,22,23,24]. They combine hydrophilic, highly degradable PLA with slow degrading, hydrophobic PLC with excellent biocompatibility [14].

To investigate the effects of the three-dimensional structure, we compared porous scaffolds (PS) based on Schwarz Primitive (P) minimal surface-derived unit cells to solid non-porous scaffolds (CS).

The impact of porosity and pore sizes on MSC and osteoblasts has been observed in multiple studies. While smaller, nano pore sizes lead to enhanced MSC cell attachment, larger pore sizes are essential for MSC osteogenic and chondrogenic differentiation and cell migration into the scaffold [16,25,26]. Porosity and pore interconnection of the scaffold also play an important role in its biocompatibility since they affect biodegradation and nutrient transport [16]. Further, chemical and physical properties, e.g., surface energy or roughness of the surface influence the osteochondral regeneration [26,27].

Several studies focused on the regeneration of cartilage. Proper cartilage is of course most important for regeneration of osteochondral defects. However, the subchondral bone is also important. Increased activity of bone resorbing osteoclasts lead to enhanced degradation of the biphasic scaffold that subsequently sinters in its height followed by formation of stairs or gaps in the articular cartilage. Therefore, we focused in this study on the bony part of the implant. Since both cell types, tissue forming osteoblasts as well as tissue degrading osteoclasts, interact in reconstruction of osteochondral defects, both must be vital on the implant [28]. Here, we analyzed the cytocompatibility of the scaffolds with MSC as progenitors of bone forming osteoblasts using lactate dehydrogenase (LDH) assay and MTT assay, which are well established methods to evaluate cytotoxicity and viability [29]. Further, we observed how the materials effect the differentiation of human peripheral blood mononuclear cells (PBMC) towards mature osteoclast and the activity of the osteoclasts. Relative gene expression of typical osteoclast differentiation and activity targets [30,31,32,33,34] as well as osteoclast-specific enzyme tartrate-resistant acid phosphatase (TRAP) isoform 5b activity [35,36,37] were measured at different time points. The present study is the first investigation of (i) the viability of human MSC as well as (ii) the differentiation of human PBMC into osteoclasts and (iii) the activity of osteoclasts in direct contact to porous scaffolds (PS) compared to compact scaffolds (CS) of LCM. In a previous study, Kampleitner et al. (2020) analyzed the proliferation of murine MC3T3 osteoblast cell line and morphology of murine osteoclasts on porous scaffolds of LCM (pore size of 314 ± 14 µm) compared to pure cell culture plastic [3]. Thus, the major novelty of our study was the analysis of human cells on porous LCM scaffolds compared to compact scaffolds. However, we also decreased the pore size from 314 to 170 µm to improve the biomechanical stability of the implant. The results of this study will be used in a research program that aims to establish biphasic polymer-based implants for osteochondral defects.

## 2. Results

### 2.1. Viability of MSC

**Figure 1:**
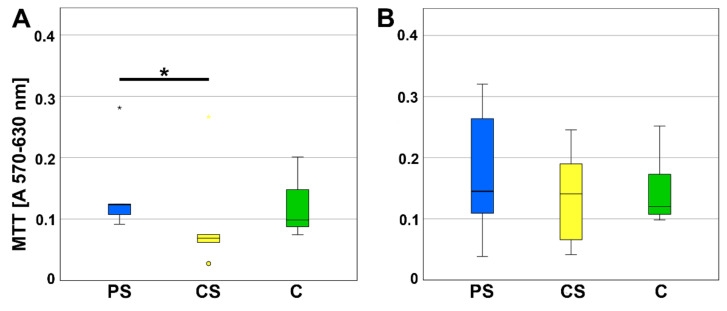
MTT assay (**A**) after 3 (d) and (**B**) 6 days of incubation. CS—compact scaffolds, PS—porous scaffolds, C—control on cell culture plastic. * indicates a statistically significant likelihood of *p* ≤ 0.05. Circles and small asterisks denote outliers.

The viability was determined by MTT assay after three and six days of culturing MSC on the scaffolds (Figure 1). On day three, a significantly better viability was measured for MSC growing on porous scaffolds compared to compact scaffolds (*p* = 0.043). After six days of culturing, no significant differences were calculated.

### 2.2. Cytotoxicity

**Figure 2:**
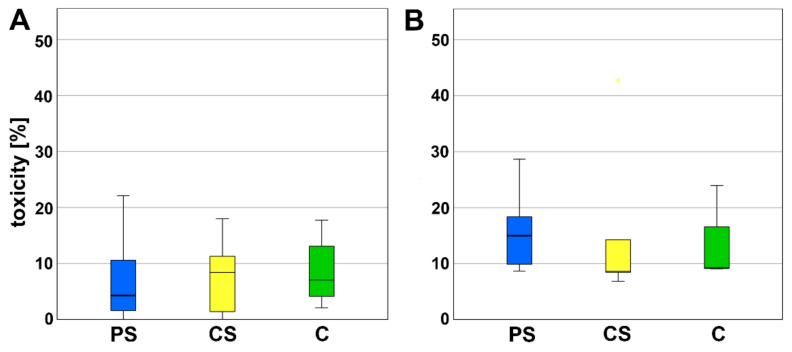
LDH assay (**A**) after 3 (d) and (**B**) 6 days of incubation. PS—porous scaffolds, CS—compact scaffolds, C—control on cell culture plastic.

The LDH content was investigated to determine the cytotoxicity. No significant alterations in LDH were measured for PS compared to CS as well as to pure cell culture plastic that was used as control (Figure 2).

### 2.3. Imaging of MSC

**Figure 3:**
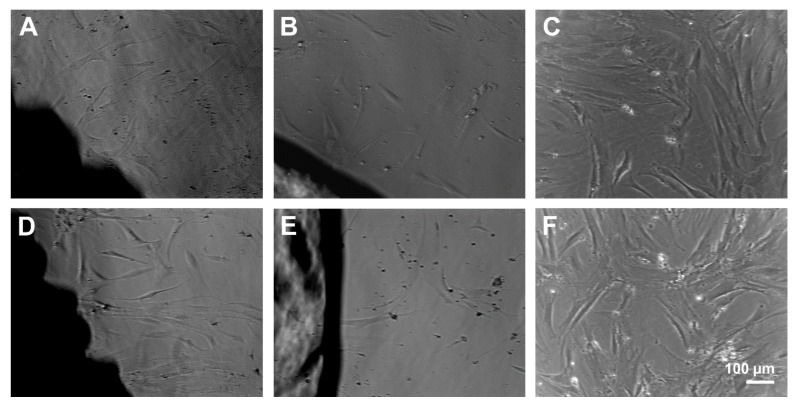
MSC growing at the surface of porous scaffolds (**A**,**D**) and compact scaffolds (**B**,**E**) for 3 (**A**–**C**) and 6 days (**D**–**F**). Scale bar of F is representative for (**A**–**E**).

Light microscopy was used to determine changes in cell morphology. No morphological changes were observed at the interface of PS and CS implants compared to the cells growing on cell culture plastic. Under all conditions, rapid self-renewing cells (RS1), typical spindle-shaped cells (RS2) and large, flattened cells were visible. The materials did not cause any rejection reaction at time points three and six days. However, MSC on cell culture plastic seemed to be superior in number and adhesion of cells compared to the materials. The enhanced cell number was most obvious for the RS1 cells. Further, fewer cells grew in presence of CS compared to PS (Figure 3).

### 2.4. Osteoclastogenesis

**Figure 4:**
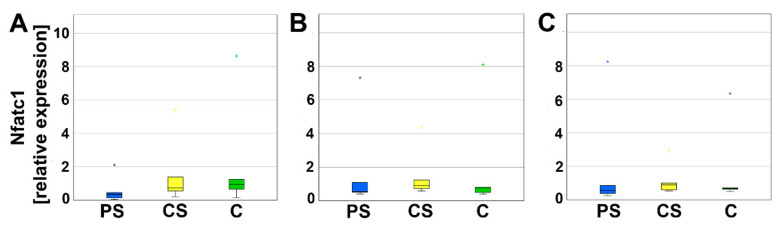
Relative expression of Nfatc1 after 4 (**A**), 8 (**B**), and 12 days (**C**). Small asterisks denote outliers.

To evaluate the differentiation of PBMC as progenitor cells to mature osteoclasts, relative expression of nuclear factor of activated t-cells (Nfatc1) and dendritic cell specific transmembrane protein (DC-Stamp) was analyzed using real-time reverse transcriptase polymerase chain reaction (real-time RT-PCR). Nfatc1 serves as a transcription factor for osteoclast-specific genes. After 4, 8, and 12 days of culturing, no significant alterations in the relative expression of Nfatc1 were detected (Figure 4).

**Figure 5:**
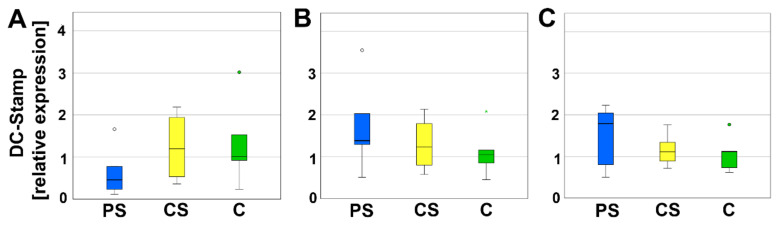
Relative expression of DC-Stamp after 4 (**A**), 8 (**B**), and 12 days (**C**). Circles and small asterisks denote outliers.

DC-Stamp is involved in the fusion of progenitor cells. Neither on day 4, nor on day 8 or 12, significant differences of relative expression between PS, CS, and controls could be measured (Figure 5).

### 2.5. Activity of Osteoclasts

**Figure 6:**
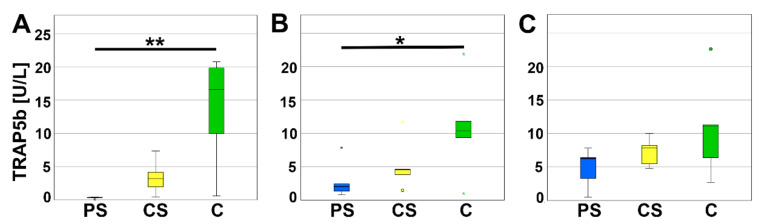
TRAP5b assay after 4 (**A**), 8 (**B**), and 12 days (**C**). * indicates a statistically significant likelihood of *p* ≤ 0.05, ** indicates a statistically significant likelihood of *p* ≤ 0.01. Circles and small asterisks denote outliers.

TRAP5b is an enzyme secreted by osteoclasts causing degradation of bone matrix and is therefore considered as a typical marker for osteoclast activity. A significant reduction of TRAP5b activity was measured for PS compared to controls after four (*p* = 0.005) and eight days (*p* = 0.034). The values of CS are located between PS and controls and were neither significant compared to the PS nor to the controls. At day 12 of cell culture incubation no significant regulation of TRAP5b activity was measured (Figure 6).

**Figure 7:**
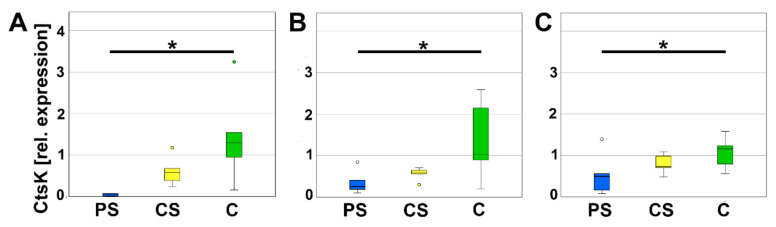
Relative expression of CtsK after 4 (**A**), 8 (**B**), and 12 days (**C**). * indicates a statistically significant likelihood of *p* ≤ 0.05. Circles denote outliers.

In addition, the mRNA-expression of osteoclast specific proteins was analyzed to evaluate osteoclast activity. Cathepsin K (CtsK) is secreted by osteoclasts and mainly involved in degradation of Collagen I. On days 4, 8, and 12, relative expression of CtsK of osteoclasts cultured on PS was significantly less compared to C. Relative expression of CtsK of CS was higher than PS, but lower than C and therefore not significantly different to either one (Figure 7).

**Figure 8:**
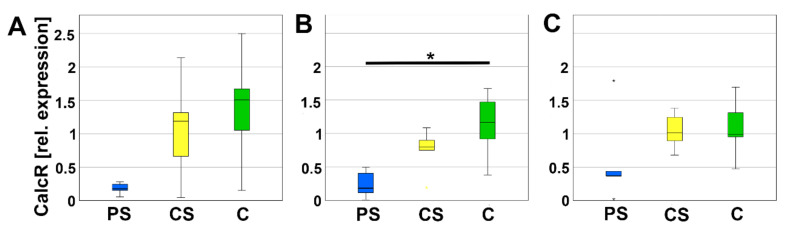
Relative expression of CalcR after 4 (**A**), 8 (**B**), and 12 days (**C**). * indicates a statistically significant likelihood of *p* ≤ 0.05. Small asterisks denote outliers.

Due to their important role in the balance, mature, active osteoclasts express a calcitonin-receptor (CalcR). On day 8, relative expression of CalcR was significantly lower on PS compared to C. On day 4 and day 12, no significant alterations were detected (Figure 8).

**Figure 9:**
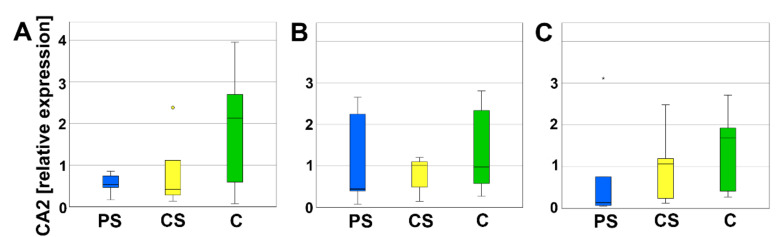
Relative expression of CA2 after 4 (**A**), 8 (**B**), and 12 days (**C**). Circles and small asterisks denote outliers.

Carbonic anhydrase 2 (CA2) is expressed by osteoclasts to ascertain the acidic milieu of the Howship’s lacunae. No significant differences of the relative expression of CA2 were measured between PS, CS, and C (Figure 9).

### 2.6. Cell Morphology and Adhesion of Osteoclasts

**Figure 10:**
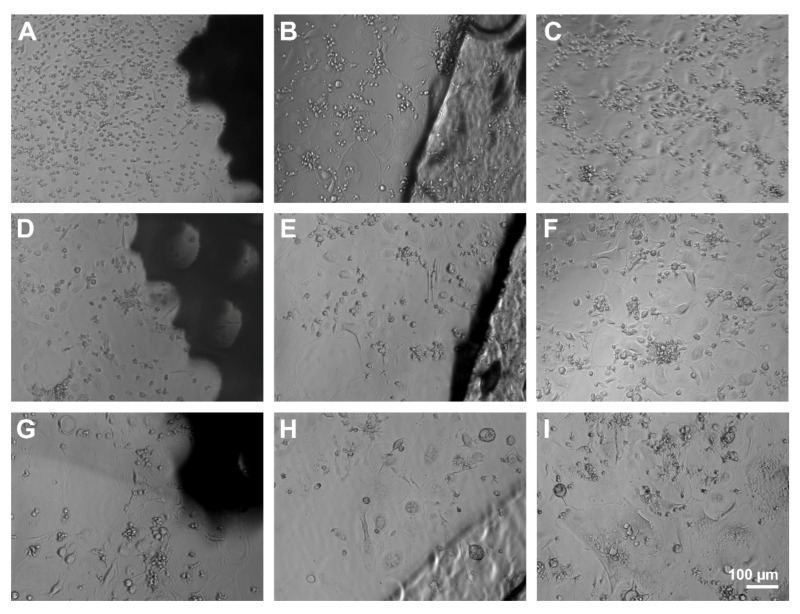
Light microscopic observation of PBMC and osteoclasts after 4 (**A**–**C**), 8 (**D**–**F**), and 12 days (**G**–**I**) at the interface of PS (**A**,**D**,**G**), CS (**B**,**E**,**H**), and pure cell culture plastic (control, **C**,**F**,**I**). Scale bar of I is representative for (**A**–**H**).

Light microscopic observation of the PBMC allowed to distinguish different cell types during the differentiation. At the early stages, mononucleated round-shaped cells with little cytoplasma represented the majority of the cells. After that, mononucleated spindle-shaped cells took over. Later, bigger mononucleated round-shaped cells and multinucleated cells appeared, accompanied by a decrease of smaller cells. Although no rejection reaction was detected at the interface between scaffolds and the cell monolayer and cells seemed to grow on top of and under the material, differentiation seemed to progress slower in the presence of the porous scaffolds. While multinucleated cells could be detected with compact scaffolds and controls without material on day four, only mononucleated round- and spindle-shaped cells displayed next to porous scaffolds. Also, fewer osteoclast-like giant cells with sizes >400 µm appeared in contact with PS compared to CS and C (Figure 10).

**Figure 11:**
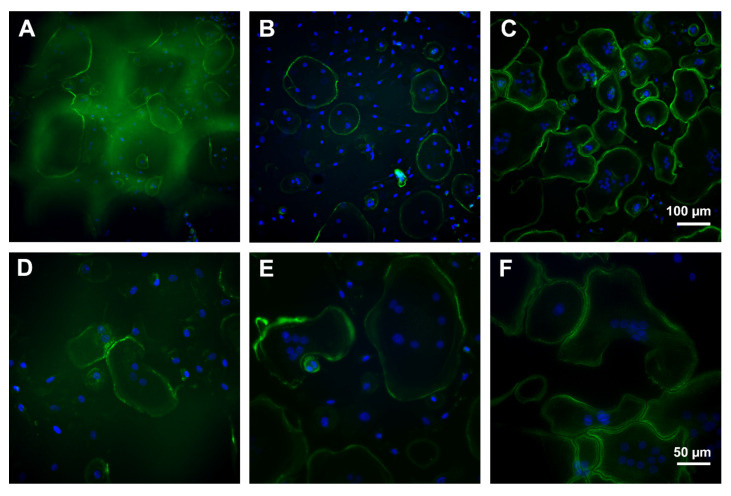
Immunofluorescence microscopy of osteoclasts after eight days of incubation on porous scaffold (**A**,**D**), compact scaffold (**B**,**E**), and cell culture plastic (**C**,**F**). Green: actin labelling, blue: nuclei. Scale bar of (**C**) is representative for (**A**,**B**) and of (**F**) for (**D**,**E**).

To assess the growth and the adhesion, PBMC were incubated for eight days and analyzed by immunofluorescence microscopy. The control culture displayed unaltered osteoclast differentiation portrayed by a monolayer dominated by large, multinuclear osteoclasts. Mature osteoclasts were characterized by podosomes organized in clusters and a typical ring formation depicted by immunolabelling of actin conjugated with fluorescein isothiocyanat (FITC, green). Further, pseudopodia were visible. In contrast, cells on PS were mostly small, mononucleated round-shaped cells. Multinucleated cells were smaller and displayed a smaller amount of nuclei compared to control. Nuclei were labelled with Hoechst 33258 stain. Cells on CS seemed to be more differentiated than on PS, but not as mature as on pure cell culture plastic (control). The monolayer was dominated by mononucleated, spindle-shaped cells, but osteoclast-like, multinucleated cells could also be detected (Figure 11).

## 3. Discussion

In the present study, we analyzed whether scaffolds with interconnected pores (PS) are more suitable for MSC and osteoclasts compared to solid implants (CS). Both scaffolds were generated from the same material: poly-((D,L)-lactide-ε-caprolactone)dimethacrylate (LCM). Previous studies demonstrated that grafts of LCM are suitable for growth of chondrocytes. Jung et al. (2008) reported enhanced deposition of chondral extracellular matrix onto LCM [38]. Kosorn et al. (2019) treated the surface of LCM grafts enzymatically and reached higher hydrophilicity and improved chondrocyte proliferation and function [39]. Here, we did not study the growth and proliferation of chondrocytes, but of MSC. MSC are the progenitors of chondrocytes as well as of osteoblasts. In the case of a traumatic lesion, it is necessary that MSC migrate into the destroyed area, proliferate, attach to the material, and remain viable. Subsequently, they will differentiate into osteoblasts or in chondrocytes as regulated by the surrounding tissue, biomechanical stability, and growth factors. We measured an increased viability of MSC at the surface of PS compared to CS early after seeding, whereas at the late time point, no significant differences were found. Since CS and PS are generated from the same material, it can be concluded that the 3D geometry is responsible for the differences in viability. Due to the 3D geometry, PS contains an augmented area of growth compared to CS. We suppose that this causes a higher proliferation rate of MSC at day three, as well as the increased metabolic activity. However, on day six, the positive effect in metabolic activity was already finished. We expect that the short time of the 3D effect is caused by the relatively small height of PS with 1 mm. However, Duval et al. 2017 already stated that the possibility to grow in 3D influences the fundamental cellular behavior depending on the cell line and the characteristics of the scaffold [40]. Thus, our results are in line with their studies on 3D cell cultures.

Biological and physicochemical properties of our scaffolds are similar to the ones investigated by Kampleitner et al. (2020) that were also generated by the Institute for Bioprocessing and Analytical Measurement Techniques (iba, Heiligenstadt, Germany). Kampleitner et al. examined the biocompatibility of Schwarz P minimal surface LCM scaffolds fabricated via two-photon polymerization with different lactide/caprolactone ratios and chain lengths [3]. The LA/PLC ratio of 16:4, which was also used in our study, appeared to be the most promising candidate since it displayed proliferation of primary murine osteoblasts, slow in vitro-degradation, as well as cellular infiltrates, angiogenesis, and sufficient stability in vivo [3]. So far, vitality of human MSC as well as human osteoclasts were not investigated. Besides MTT, we measured the LDH content to determine cytotoxic effects of MSC. No significant differences were detected for MSC, growing on LCM compared to the controls growing on tissue culture plastic. Thus, our results verify the already described excellent cytocompatibility of LCM with an additional cell type, human MSC.

Live cell imaging by means of light microscopy demonstrated that the MSC attached at the LCM scaffolds hold the typical morphology of large, slowly replicating cells as well as the rapidly dividing, spindle-shaped cells. These are two of the typical morphological phenotypes described for MSC [41]. The third cell type are so called rapid self-renewing (RS) cells that are very small (7 µm) and rapidly dividing [41]. These cells were also observed in all three groups. However, in the controls, the amount of RS was higher than in the PS and CS group. Thus, we suppose that MSC in plastic control have a high proliferation rate. Surprisingly, it is not higher than in cells at the PS. Besides, no significant changes were observed in the MSC morphology.

Regarding scaffold degradation, Kampleitner et al. 2020 reported that all LCM scaffolds altered the morphology of murine osteoclast compared with control osteoclast cultured without material. Fewer osteoclasts were found and they showed a decrease in size when growing on LCM scaffolds [3]. The degradation without cells was previously analyzed by rates of mass loss [17]. At the temperature of 37 °C, 20% of the scaffold is degraded after 90 days of incubation [17]. The effect of osteoclasts on the degradation process of LCM scaffolds is not known.

For our analysis on osteoclasts, we used the material (LCM3 16:4) as described in Kampleitner et al. (2020) [3]. Our results on the differentiation of human PBMC in osteoclasts confirm the observations of Kampleitner et al. on murine osteoclasts. We found smaller osteoclasts at the interface of PS. Interestingly, the cells at the CS showed a more advanced differentiation into osteoclast than at the PS. Therefore, progress of osteoclastogenesis at CS was more similar to the controls than at PS scaffolds. Thus, we suppose that the delayed osteoclastogenesis is not caused by the LCM alone, but by the combination of material and spatial architecture of the scaffolds.

Zhang et al. analyzed murine osteoclasts differentiated from the murine cell line RAW264.7 [36]. He observed osteoclasts with significantly larger and more heterogenous F-actin rings on smooth surface compared with rough surfaces. F-actin rings are indicators for attachment of osteoclasts [38]. In the present study, F-actin rings were visible under all conditions. Thus, the osteoclasts attached well at the surface of LCM equal of the 3D geometry.

In addition, Zhang et al. reported a significantly higher number of nuclei per osteoclast cultured on smooth surfaces compared to rough surfaces [42]. We observed that most cells of our osteoclast differentiation culture on PS were small and mononucleated. This might indicate that the osteoclasts behave on the PS like they do on rough surfaces. In addition, a few of the multinucleated cells at PS were also smaller and displayed a lower number of nuclei compared to cells on CS and plastic control. Thus, from the morphological view, we suppose that the osteoclastogenesis is retarded after eight days of incubation.

Real-time RT-PCR was performed for transcription factors of the osteoclastogenesis to verify the implications of morphology. DC-STAMP was investigated because it mediates the fusion of mononuclear osteoclast precursors [42]. Since less multinucleated cells were found by means of immunofluorescence, we expected a decline in its mRNA expression. However, no significant changes were determined for DC-STAMP mRNA expression. Regulations in mRNA expression are often situated before protein is regulated. Thus, the decrease in mRNA expression might be finalized at the harvesting time point on day eight and not be started at day four. In future studies, the gap between the investigation time points will be reduced. Besides DC-STAMP, several other factors are involved in the fusion of preosteoclasts, e.g., Atp6v0d2 [43]. As second target for the osteoclastogenesis, we analyzed Nfatc1 mRNA expression. Nfatc1 is an essential transcription factor in osteoclastogenesis. It is induced, e.g., by receptor activator nuclear factor kappa B ligand (RANKL) pathway, and acts on his own gene by an autoamplification loop [44]. The effect of LCM on Nfatc1 expression was not analyzed so far. Inamitsu et al. (2016) reported that Nfatc1 expression was increased by monomers with different compositions of methacrylate released by a dental resin matrix [45]. Thus, synthetic molecules like polymers are able to influence the osteoclastogenesis by regulation of Nfatc1 expression. Here, we did not detect a regulation in Nfatc1 expression of the osteoclast progenitor cells situated in direct contact with LCM. In addition to DC-STAMP and Nfatc1, we also investigated CalcR, which is a marker for mature osteoclasts as well a marker for the activity of osteoclasts. It is localized at the basolateral membrane of osteoclasts. If calcitonin binds, the osteoclasts stop resorption and migrate from the bone matrix [46]. At day eight, we measured a significant decrease in CalcR expression at PS compared to the controls. This might be a hint for a delay in osteoclastogenesis. On the other side, it might also indicate a reduced activity of osteoclasts.

Important enzymes for the activity of osteoclasts are CtsK and CA2. On mRNA level, we did not detect regulation of CA2. CA2 is involved in the H^+^ production. H^+^ release in the resorption lacunae (howship lacunae) is necessary for the degradation of the inorganic bone matrix [46]. It can be argued that a further pH regulation is not necessary because during degradation, LCM produces lactic acid and therefore the pH of Howship’s lacunae is acidified.

In addition, we investigated CtsK. In line with Zhang et al., 2018 [42], we measured a reduced gene expression of CtsK at all investigated time points. CtsK belongs to the family of cysteine proteases and cleaves Gly-Gly bonds. Hence, it is involved in the degradation of organic bone matrix. Besides CtsK, TRAP5b is an important enzyme for the degradation of organic bone matrix. At the PS, we observed a reduction in TRAP5b activity by means of a functional assay. The decline in TRAP activity is in accordance with the studies of Jones et al. [47] and Zhang et al. [42]. Jones et al. analyzed the TRAP activity by enzyme histochemical staining and observed a low TRAP activity combined with a supported osteoblast proliferation and attachment of cells cultured to poly-l-lactic acid (PLLA) films for ten days [47]. This is highly similar to our observations at the porous LCM scaffolds. Zhang et al. measured a reduced TRAP activity in osteoclasts growing at rough surfaces compared to smooth surfaces [42]. We suppose that the surface of PS provokes similar effects like rough surfaces including the reduction in osteoclastic activity. Osteoclasts are important for proper bone remodeling because they stimulate the activity of osteoblasts via several mechanism (e.g., the release of insulin-like growth factor 1 (IGF1) and transforming growth factor-beta 1 (TGF-β1) [48]) and are involved in the conversion of woven bone in the biomechanical enhanced lamellar bone. Thus, it is a sign of cytocompatibility that monocytes seeded on PS were able to differentiate in healthy osteoclasts. However, insertion of implants often leads to an increased osteoclast size, multinuclearity, number, and activity. Sometimes the cells transform into foreign-body giant cells. Overstimulation of osteoclast activity results in rapid degradation often before bony consolidation and is followed by sintering of the scaffold. Thus, we suppose, that it is a good sign for PS that the activity of osteoclasts is reduced, but not inhibited. Beside the roughness, also the relatively small pore size with 170 µm might be engaged in the decline in osteoclast activity.

Large pore size and high porosity lead to faster degradation of the scaffolds [16,49], but also favors the ingrowth of the MSC as progenitors of bone and cartilage. Requirements for biodegradable implants are an appropriate degradation rate that allows the cartilage and bone forming cells to replace the naturally produced tissue. The LCM scaffolds seem to fulfill this request well [14,17].

Furthermore, the implants have to be cytocompatible. They need to allow good cellular attachment, proliferation, and should not be cytotoxic. All these requirements are given (Figure 1, Figure 2 and Figure 3) for the analyzed implants, especially for the PS. Schwarz P unit cells were used as minimal structural units for fabrication of PS. They provide high porosity, interconnectivity, and high fluid permeability that allows a sufficient transport of nutrient, oxygen, and metabolic waste [14]. To print the Schwarz P unit cells, the polymers were photo-crosslinked by two-photon polymerization (2PP), which enabled defined shape and size combined with high reproducibility and tunable mechanical properties [14].

## 4. Materials and Methods

### 4.1. Scaffolds

**Figure 12:**
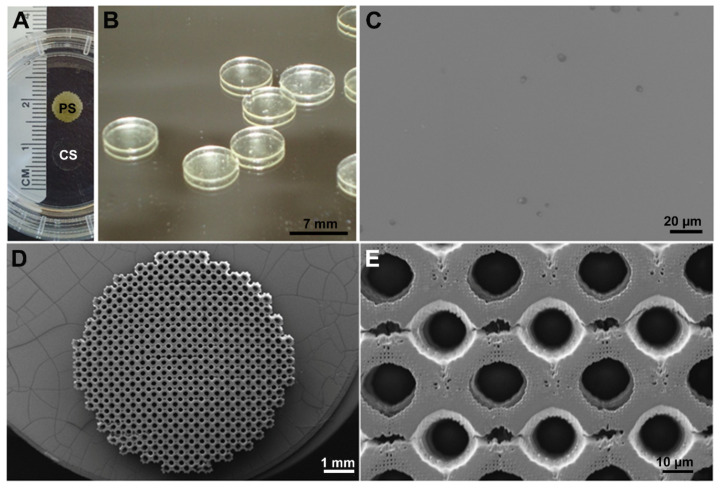
Images of porous (PS) and compact LCM scaffolds (CS). (**A**) Overview of PS compared to CS that is nearly transparent. (**B**) Higher magnification of CS. (**C**) Scanning electron microscopic (SEM) picture of the surface of CS. (**D**) SEM overview of PS. (**E**) Detail SEM of PS.

The scaffolds were developed and produced by IBA Heiligenstadt (Department of Biomaterials, Institute for Bioprocessing and Analytical Measurement Techniques e.V., Germany) with the dimension of 7 mm in diameter and 1 mm in height. They were generated with biodegradable poly-((D,L)-lactide-ε-caprolactone)dimethacrylate (LCM) via 2-PP (porous scaffolds) or UV-curing (solid scaffolds) in a ratio of 8:4 ((D,L)-lactide: ε-caprolactone) as described previously [3,17]. Solid (compact) scaffolds (CS) were compared with porous scaffolds (PS) that consisted of Schwarz-P-cells. Schwarz-P-minimal surface units provide a high surface-to-volume ratio. The used Schwarz-P-cell is characterized by an elastic modulus of 0.16 MPa, a porosity of 0.937, and a pore size of 170 µm. The scaffolds were examined by scanning electron microscopy (SEM, JEOL 6400) (Figure 12). The implants were stored in ethanol and thoroughly washed in water, which was renewed thrice a day for two days before being used.

### 4.2. Cell Culture of MSC

Human MSC were harvested from reaming debris and femoral heads from patients (*n* = 5) that underwent routine surgery (e.g., insertions of an endoprosthesis) in the Department of Trauma Surgery of the University Hospital of Giessen and Marburg GmbH (UKGM) at the Campus Giessen. The study was approved by the local Ethics Commission (Reference number: 74/09) and the patients gave written informed consent before participating in this study. The group of donors included 4 females and 3 males and the mean age was 62.1 ± 19.9. None of them displayed any clinical condition related to bone metabolism.

Briefly, spongy bone fragments were washed carefully in phosphate buffered saline (PBS, Gibco, Grand Island, NY, USA) and preincubated for 10 min at 37 °C in Hibernate A (Gibco) with 10% fetal bovine serum (FBS, PAN Biotech, Aidenbach, Germany). Then, samples incubated in digested solution consisting of Hibernate A, 0.1 M/mL calcium chloride and 10 µg/mL of collagenase (Fujifilm Wako, Neuss, Germany) for 60 min at 37 °C on a shaking device. Digestion was stopped by Hibernate A with 10% FBS MSC (PAN Biotech). The solution was centrifuged and the pellet was diluted in erythrocyte-lysis-buffer consisting of 77.5 mM/mL ammonium chloride, 5 mM potassium bicarbonate, and 5 nM/mL EDTA. Subsequently, 5 mL of Hibernate A with 10% FBS were added, the solution was filtered with a 70 µm cell sieve and centrifuged for 5 min at 4 °C and 500 g. The pellet was carefully washed with Hibernate A with 10% FBS. Then, Fluorescence-activated Cell Sorting was performed using a BD FACS Aria II FACS sorter (BD Biosciences, San Jose, CA, USA) and the following antibodies: (a) PB-conjugated mouse-anti-human CD73 (BioLegend, San Diego, CA, USA), (b) APC-conjugated mouse-anti-human CD105 (BioLegend), (c) mouse-anti-human CD31-APC/Cy7 (BioLegend), and (d) mouse-anti-human CD45-APC/Cy7 (BioLegend). The sorted MSC fraction was cultured in MesenPro RS medium (Gibco) with 10% FBS (PAN Biotech), 0.25 µg/mL amphotericin B, 10 µg/mL gentamycin (Gentamycin/Amphotericin B, ThermoFisher Scientific, Waltham, MA, USA), and 1% Glutamine (Capricorn Scientific GmbH, Ebsdorfergrund, Germany).

MSC were characterized by positive binding to CD73 and CD105, negative binding to CD31 and CD45 as well as by plastic adherence for at least 10 passages and differentiation into osteoblasts, chondrocytes, and adipocytes.

### 4.3. Cell Viability Assay

For the determination of cell viability, 15,000 cells were seeded into 48-well plates on which there was each one well with PS, CS, and control. Culture medium consisted of Dulbecco’s Modified Eagle Medium (DMEM) low glucose (Gibco), 10% FBS MSC (PAN-Biotech), 1% Gentamycin/Amphotericin B (ThermoFisher Scientific), and 1% Glutamine (Capricorn Scientific GmbH). To estimate the metabolic activity of the cells, MTT (3-[4,5-dimethylthiazol-2-yl]-2,5 diphenyl tetrazolium bromide, Sigma-Aldrich Chemie GmbH, St. Louis, MO, USA) colorimetric assay was performed at the time points 3 and 6 days (d3, d6). Therefore, 50 µL MTT solution were added to the cell medium. The cells were then incubated at 37 °C for 4 h in the dark. Subsequently, the medium was discarded and 0.04 N hydrocloridric acid in 2-Propanol was added to lyse the cells, followed by 10 min of shaking on ice. The cell lysates were transferred as couplets to a 96-well plate for the absorbance measure at 570 nm and 630 nm using a Synergy HT microplate Reader (BioTek, Bad Friedrichshall, Germany).

### 4.4. Cytotoxicity Assay

To ascertain the cytotoxic effects of LCM scaffolds on MSC, the cells were seeded and cultured equally, as for MTT assay. Likewise, the time points of investigation were 3 and 6 days (d3, d6). The cytotoxicity was estimated based on determination of the concentration of lactate dehydrogenase (LDH) by using the Cytotox 96^©^ Non-Radioactive Cytotoxicity Assay (Promega, Fitchburg, MA, USA). First, 50 µL of the cell medium were transferred to a 96-well plate. Second, the medium was discarded and 1% triton-X 100 (Sigma) in PBS was added to disrupt the cell membrane and release LDH. After 15 min of shaking on ice, the plate was centrifuged at 500 g and 4 °C for 5 min. The supernatants were also transferred to the 96-well plate. Next, 50 µL LDH substrate were added to each sample followed by 30 min of incubating in the dark at room temperature. Finally, 50 µL of stop solution were added to the wells before measuring absorbance at 490 nm using a Synergy HT microplate Reader (Biotek). The assay was performed in triplets. The relative cytotoxicity was calculated from the quotient of LDH_medium_/LDH_total_ (LDH_medium_ + LDH_lysate_) and transformed into percent for the graph (Figure 2).

### 4.5. Life Cell Observation of MSC

Cells were observed using a Zeiss Axiovert 10 microscope (Zeiss, Oberkochen, Germany) equipped with a Stingray F-145 camera (Allied Vision Technologies GmbH, Stadtroda, Germany) on days 3 and 6.

### 4.6. Osteoclast Cell Culture System

To investigate the effects of the material on osteoclast differentiation and activity, monocytes were isolated from human peripheral blood mononuclear cells (PBMC) from five anonymous donors (*n* = 5), which were provided by the Institute of Clinical Immunology and Transfusion Medicine (Justus Liebig University, Giessen, Germany). The usage of donor blood was permitted by the local Ethics Commission (Reference number: 05/00) and donors gave written informed consent before participation. In detail, 10 mL blood were diluted in 25 mL 4 °C tempered PBS (Gibco) including 2% fetal bovine serum (FBS, Biochrom, Berlin, Germany) and 1 mM ethylendiaminetetraacetic acid (EDTA). Following this, the dilution was transferred into a Leucosep centrifuge tube (Greiner Bio-One, Frickenhausen, Germany) for density gradient centrifugation for 15 min at 800 g without activating the brake. White blood cells were obtained, washed with 50 mL cold PBS and centrifuged for 5 min at 450 g before 30 mL cold PBS were added and centrifuged for 10 min at 120 g. After that, 30 mL PBS were added and centrifuged again for 10 min at 120 g. Consequently, PBMCs were resuspended in 1 mL PBS and the number of cells was determined using the CASY cell counter (model TTC, Omni life sciences GmbH, Bremen, Germany). For isolation of monocytes, PBMCs were diluted in PBS to a concentration of $5\times 10^{7}$ cells/mL. A total of 2 mL of cell dilution were shaken with 50 µL of the EasySep^TM^ Human Monocyte Enrichment Kit (Stemcell Technologies, Vancouver, Canada) and incubated for 10 min at 2–8 °C. The kit contains an antibody complex that recognizes non-monocyte cells and dextran-coated magnetic particles. Next, 50 µL of EasySep^TM^ Magnetic Particles (Stemcell Technologies) were added, shaken, and incubated for 5 min at 2–8 °C. Afterwards, 300 µL PBS were admixed and the tube was placed in an EasySep^TM^ magnet (Stemcell Technologies) for 2.5 min at room temperature. Non-monocyte cells are adhered to the magnet and the monocytes were transferred to a new 50 mL falcon and centrifuged for 5 min at 450 g. Supernatant was decanted, cells were resuspended in 1 mL PBS, and the number of cells was determined using CASY cell counter. Monocytes are characterized by negative labelling of CD16 and positive labelling of CD14. A total of 200,000 monocytes were resuspended in 500 µL of medium. The scaffolds were carefully washed for 48 h in distilled water that was changed 3 times daily. Then, they were placed into a 48-well plate and covered with 500 µL of medium. Finally, 200,000 monocytes were seeded on top of the scaffolds. Triplicates were performed for every condition. Composition of the medium was alpha modified essential medium (αMEM, Gibco) supplemented with 10% FBS (Biochrom, Berlin, Germany), 0.25 µg/mL amphotericin B, 10 µg/mL gentamycin (ThermoFisher Scientific), 1% Glutamine (Capricorn Scientific GmbH), as well as 50 ng/mL MCSF (Sigma-Aldrich) and 50 ng/mL RANKL (Santa Cruz Biotechnology Inc., Dallas, TX, USA). Monocytes were incubated at 37 °C and 5% CO_2_ and medium was renewed every other day.

### 4.7. Real-Time Reverse Transcriptase Polymerase Chain Reaction (Real-Time RT-PCR)

To isolate osteoclast RNA, RNeasy^®^ Mini Kit (Qiagen GmbH, Hilden, Germany) was used according to the protocol of the manufacturer. Briefly, the cells were washed with PBS (Gibco) before being lysed with 250 µL RLT-Buffer (kit component). The solution was loaded on QIAshredder column (Qiagen) and centrifuged to homogenize the lysate. Thereafter, the through flow was transferred to RNeasy Mini spin column (kit component) and carefully washed. Then, RNA was diluted in 30 µL RNase-free water and the RNA concentration was determined using NanoDrop spectrometry (ND-1000, Thermo Fisher Scientific). In the next step, QuantiTect^®^ Reverse Transcription Kit (Qiagen) was used to synthesize cDNA. First, Wipeout-Buffer (kit component) was added to eliminate genomic DNA and the solution was incubated for 5 min at 42 °C. Second, a mastermix consisting of Quantiscript reverse transcriptase, buffer, and primers (all kit components) were added before being incubated at 42 °C for 30 min. Finally, the solution was heated to 95 °C for 3 min to inactivate reverse transcriptase. Efficiency of gene-specific primers (Table 1, Eurofins Genomics, Ebersberg, Germany) was controlled by serial dilutions and standard curve. The mRNA-expression of the genes was analyzed using a LightCycler^®^ 2.0 instrument (Roche, Basel, Switzerland) for real-time RT-PCR under the following conditions: For beta-2-microglobulin (B2M), cathepsin K (CtsK), nuclear factor of activated t-cells (Nfatc1), dendritic cell specific transmembrane protein (DC-STAMP), and calcitonin receptor (CalcR) QuantiFast^®^ SYBR^®^ Green PCR Kit (Qiagen) was used. The cycling procedure consisted of 5 min of heating at 95 °C followed by 40 cycles of each 10 s at 95 °C for denaturation, annealing for 30 s at 60 °C and subsequent melting curve to verify the purity of the RT-PCR products. LightCycler^®^ FastStart^PLUS^ DNA Master SYBR^®^ Green I (Roche) was used for analysis of the gene expression of carbonic anhydrase 2 (CA2) and the following program: 10 min initial denaturation at 95 °C, 40 cycles with 5 s at 95 °C for denaturation, and 30 s at 64 °C for annealing, followed by melting curve. Negative controls were performed with samples that were not reverse transcribed or received water instead of cDNA. Results were calculated using 2^−∆∆CT^ method [50], while housekeeping gene B2M served as reference. Relative gene expressions were related to the median value of controls at the corresponding time of culturing.

### 4.8. Tartrate-Resistant Acidic Phosphatase (TRAP) 5b Enzyme-Linked Immunosorbent Assay

For determination of TRAP5b activity, sample supernatants of osteoclast cell culture were collected at the time points 4, 8, and 12 days (d4, d8, d12). Triplicates were performed for each condition. The Microvue TRAP5b ELISA Kit (Quidel, San Diego, CA, USA) was used as described in the manufacturer’s manual. In brief, 50 µL dilution buffer (kit component) were mixed with 50 µL cell culture supernatant for 60 min at room temperature and 300 U/min. Following this, wells were washed thrice using wash buffer (kit component). After incubation with 100 µL substrate (kit component) for 60 min at 37 °C, 50 µL stop solution (kit component) were added. The absorbance was measured using a Synergy HT microplate Reader (Biotek) at 405 nm.

### 4.9. Life Cell Obserevation of Osteoclasts

Changes in outer cell morphology were observed using a Zeiss Axiovert 10 microscope (Zeiss) equipped with a Stingray F-145 camera (Allied Vision Technologies GmbH) after 2, 4, 6, 8, 10, and 12 days of incubation.

### 4.10. Immunofluorescence of Osteoclasts

To evaluate the osteoclast differentiation in contact with the different materials, the cells were examined by immunofluorescence microscopy. Therefore, human PBMCs were obtained as described above and 100,000 monocytes per well were seeded into a 96-well SCREENSTAR plate (Greiner Bio-One). Due to smaller wells, PS and CS were quartered. After being cultured as described above for 8 days, the samples were washed with PBS and fixed with 4% paraformaldehyde in PBS, pH 7.3, for 10 min. To permeabilize the cell membrane, samples were incubated for 5 min with 0.1% Triton X-100. Afterwards, 5 µg/mL fluorescein isothiocyanat (FITC)-conjugated phalloidin (Sigma-Aldrich Chemie GmbH) were added and incubated for 40 min in the dark. Following this, the samples were washed with PBS and nuclei were stained with 100 µg/mL Hoechst 33258 (Sigma-Aldrich Chemie GmbH) for 15 min in the dark. Finally, samples were mounted with ProLong Gold Antifade Reagent (Invitrogen, Eugene, OR, USA) and stored at 4 °C. Microscopy was done using a THUNDER Imager 3D Live Cell System (Leica Microsystems, Wetzlar, Germany).

### 4.11. Statistical Analysis

Statistical analysis as well as generation of graphs were performed using the SPSS statistics program (versions 26 and 27, SPSS Institute Inc., Chicago, IL, USA). All results were tested for normal distribution using Kolgomorov–Smirnov-test. Results that were not normal distributed (results of MTT-assay, LDH-cytotoxicity assay, TRAP5b ELISA, and real-time RT-PCR of CtsK and CA2) were further analyzed with the Kruskal–Wallis and Friedman test with subsequent Bonferroni correction. MTT assay results were also tested using Wilcoxon Rank Sum Test. PCR results of CTR, Nfatc1, and DC-Stamp displayed normal distribution and were therefore tested using general linear model (Multivariate test with Mauchly’s test of sphericity followed by greenhouse-geisser adjustment and huynh-feldt correction). Values of *p* ≤ 0.05 were considered statistically significant. Box plots were used as graphs where the median is depicted as bold line within the box. Data beyond three standard deviations are shown as small circles and extreme outliers as asterisks.

## 5. Conclusions

The presented results demonstrate that porous LCM scaffolds were not cytotoxic. Human MSC possess increased metabolic activity early after seeding. Human monocytes differentiated into active osteoclasts in direct contact with the scaffolds. Osteoclastic activity is restricted compared to pure cell culture plastic. Thus, a precocious degradation of the implant can be excluded. Future studies are required to analyze the differentiation potential of MSC into osteoblast and chondroblast at the interface of the implant.

## Figures and Tables

**Table 1:** Human primers used for real-time RT-PCR analysis.

Primer	Sequence	Length [bp]	Accession No.
B2M	for: 5′-TCT CTC TTT CTG GCC TGG AG-3′ rev: 5′-CAA CTT CAA TGT CGG ATG GA-3′	135	NM_004048.4
CtsK	for: 5′-AGG AGA TAC TGG ACA CCC AC-3′ rev: 5′-CCC AAA TTA AAC GCC GAG AG-3′	96	NM_000396.4
DC-Stamp	for: 5′-GCA AAG GGG AAG TCC TGA GC-3′ rev: 5′-GCC CTG CAA AGG CAA GTA G-3′	93	NM_030788.4
Nfatc1	for: 5′-GAT GGA CTG GCC GTT CTA GG-3′ rev: 5′-CGA AAT GGC GGG ATC TCA ACC-3′	70	NM_172390.3
CA2	for: 5′-GAT GGA CTG GCC GTT CTA GG-3′ rev: 5′-TGA AGT CAG CAC TCT TGC CC-3′	115	NM_000067
CalcR	for: 5′-TGA GTG TGG AAA CCC ATT TGC-3′ rev: 5′-ATT TTG GTC ACA AGC ACC CG-3′	109	NM_001164737

for: forward primer; rev: reverse primer.

## Data Availability

Data are available from the authors.

## Sample Availability

Samples of the compounds are available from the authors.
